# Blood pressure control and treatment adherence in hypertensive patients with metabolic syndrome: protocol of a randomized controlled study based on home blood pressure telemonitoring *vs*. conventional management and assessment of psychological determinants of adherence (TELEBPMET Study)

**DOI:** 10.1186/1745-6215-14-22

**Published:** 2013-01-23

**Authors:** Gianfranco Parati, Stefano Omboni, Angelo Compare, Enzo Grossi, Edward Callus, Achille Venco, Maurizio Destro, Giuseppe Villa, Paolo Palatini, Enrico Agabiti Rosei, Simonetta Scalvini, Stefano Taddei, Dario Manfellotto, Stefano Favale, Carmine De Matteis, Michele Guglielmi

**Affiliations:** 1 Department of Cardiology, IRCCS Ospedale San Luca, Istituto Auxologico Italiano and Department of Clinical Medicine and Prevention, University of Milano Bicocca, Milano, Italy; 2 Italian Institute of Telemedicine, Varese, Italy; 3 Department of Human Sciences, University of Bergamo, Bergamo, Italy; 4 Scientific Advisor, Centro Diagnostico Italiano, Milan, Italy; 5 Department of Pediatric Cardiology & Adult with Congenital Heart Centre, IRCCS Policlinico San Donato, Milan, Italy; 6 Medicina Generale II, Centro Ipertensione, University of Insubria, Ospedale di Circolo, Varese, Italy; 7 General Medicine Unit, Treviglio-Caravaggio Hospital, Medical Department AO, Treviglio, Italy; 8 Divisione di Nefrologia ed Emodialisi, IRCCS Fondazione Salvatore Maugeri, Istituto Scientifico di Pavia, Pavia, Italy; 9 Istituto di Clinica Medica IV, Policlinico Universitario, University of Padova, Padova, Italy; 10 Clinica Medica, University of Brescia, II Medicina Generale, A.O. Spedali Civili di Brescia, Brescia, Italy; 11 Servizio Autonomo di Telemedicina, IRCCS Fondazione Salvatore Maugeri, Lumezzane, Brescia, Italy; 12 Dipartimento di Medicina Interna, Azienda Ospedaliera Universitaria di Santa Chiara, University of Pisa, Pisa, Italy; 13 Ospedale S Giovanni Calibita Fatebenefratelli, Roma, Italy; 14 U.O. Cardiologia, Azienda Ospedaliera Policlinico, University of Bari, Bari, Italy; 15 U.O.S.D. Servizio di Prevenzione e Riabilitazione Cardiopatico, Centro di Prevenzione Malattie Cardiovascolari, Presidio Ospedaliero San Felice a Cancello, San Felice a Cancello, Caserta, Italy; 16 Casa di Cura Tortorella, Salerno, Italy

**Keywords:** Hypertension, Blood pressure, Home blood pressure telemonitoring, Adherence, Anxiety, Depression, Personality traits

## Abstract

**Background:**

Inadequate blood pressure control and poor adherence to treatment remain among the major limitations in the management of hypertensive patients, particularly of those at high risk of cardiovascular events. Preliminary evidence suggests that home blood pressure telemonitoring (HBPT) might help increasing the chance of achieving blood pressure targets and improve patient’s therapeutic adherence. However, all these potential advantages of HBPT have not yet been fully investigated.

**Methods/design:**

The purpose of this open label, parallel group, randomized, controlled study is to assess whether, in patients with high cardiovascular risk (treated or untreated essential arterial hypertension - both in the office and in ambulatory conditions over 24 h - and metabolic syndrome), long-term (48 weeks) blood pressure control is more effective when based on HBPT and on the feedback to patients by their doctor between visits, or when based exclusively on blood pressure determination during quarterly office visits (conventional management (CM)). A total of 252 patients will be enrolled and randomized to usual care (*n*=84) or HBPT (*n*=168). The primary study endpoint will be the rate of subjects achieving normal daytime ambulatory blood pressure targets (<135/85 mmHg) 24 weeks and 48 weeks after randomization. In addition, the study will assess the psychological determinants of adherence and persistence to drug therapy, through specific psychological tests administered during the course of the study. Other secondary study endpoints will be related to the impact of HBPT on additional clinical and economic outcomes (number of additional medical visits, direct costs of patient management, number of antihypertensive drugs prescribed, level of cardiovascular risk, degree of target organ damage and rate of cardiovascular events, regression of the metabolic syndrome).

**Discussion:**

The TELEBPMET Study will show whether HBPT is effective in improving blood pressure control and related medical and economic outcomes in hypertensive patients with metabolic syndrome. It will also provide a comprehensive understanding of the psychological determinants of medication adherence and blood pressure control of these patients.

**Trial registration:**

Clinical Trials.gov: NCT01541566

## Background

The prevalence of arterial hypertension in the European population is estimated to approximate 20% [1]. Hypertension is one of the major risk factors for developing cardiovascular diseases such as heart failure, stroke, coronary heart disease, and renal failure, and it is one of the most frequent reasons for access to medical care [1,2]. Hypertension is also often associated with other cardiovascular risk factors such as obesity, dyslipidemia, impaired glucose tolerance, or diabetes, which give rise to a condition known as metabolic syndrome, affecting one-quarter of the population and increasing by five- to six-fold cardiac morbidity and mortality as compared to healthy individuals [3-6].

Although numerous studies have shown that antihypertensive treatment may reduce cardiovascular risk and despite availability of several valuable antihypertensive drug options, today, no more than 30% of treated hypertensive patients maintain a satisfactory blood pressure control [1,7].

This may be caused by many factors, among which poor or complete lack of adherence to treatment prescriptions have been reported to play a major role. Compliance to therapy seems to be related to many factors including the high number of pills to be taken daily, an excessive cost of the available medications, the lack of motivation and poor patient’s involvement in the management of this clinical condition, the long wait in the doctor’s office, the absence of symptoms, the inability of the patient to understand the real long-term consequences of high blood pressure, and finally the psychological and personality traits of the patients [8].

Different solutions may be adopted in order to improve blood pressure control and patients’ adherence to therapy. An effective organization for a better management of this condition based on new technologies, through which the efficacy of therapy can be monitored, could help patients to get more involved in the daily care of their treatment, and to better cooperate with the physician. One of the methods used to obtain a better therapy adherence and therefore a more effective blood pressure control is now represented by self-measurement of blood pressure at home by automatic electronic devices [9]. This method represents a valuable alternative to conventional blood pressure measurement by physicians in their offices. Several studies have shown that, compared to office blood pressure measurement, self-measurement of blood pressure at home allows a better control of blood pressure [10,11] and that the blood pressure assessment so obtained has a superior prognostic value [12-15]. Home blood pressure may also have some positive effect on therapeutic adherence, though evidence on this aspect is controversial, with some studies showing a clear benefit and others showing no advantage [16-19]. Lack of definite evidence might be attributed to the limited number of studies performed so far, and to the heterogeneity of the methodologies employed to assess and quantify patients’ compliance with treatment in the different trials [16-19].

Evidence from randomized studies suggests that home blood pressure telemonitoring (HBPT), defined as home blood pressure monitoring coupled with telematic data transmission to the doctor and real-time feedback on patient’s status, may improve blood pressure control and compliance to treatment, and may help in optimizing the patient’s therapeutic regimen [20,21].

Although HBPT may in particular be of great advantage for those patients requiring a closer follow-up, as in the case of high-risk hypertensive patients, at the moment it has not yet been clarified whether this approach may actually improve blood pressure control and adherence and persistence to antihypertensive drug treatment also in this category of hypertensive patients.

## Methods

### Study objectives

The study aims at investigating whether in high-risk patients with hypertension and metabolic syndrome, daytime ambulatory blood pressure control over 48 weeks is more effective when based on regular HBPT (intervention group), through monthly automated blood pressure teletransmission and feedback to the patient by the doctor, rather than when based only on periodic measurements made by doctors in their office during quarterly visits (conventional management (CM) control group). The main study endpoint will be the assessment and comparison of the rate of subjects achieving a daytime ambulatory blood pressure normalization (average daytime blood pressure <135/85 mmHg) in the two randomization groups (intervention *vs*. control).

The study will also assess the psychological determinants of adherence and persistence to drug therapy and the influence of HBPT on such psychological factors, as well as the impact of HBPT on additional clinical and economic outcomes. These will include the number of additional medical visits, direct costs of patient management, number of drugs or drug combinations used to achieve blood pressure control, the level of cardiovascular risk, the degree of target organ damage and frequency of cardiovascular events, and the regression of the metabolic syndrome.

### Study design

**Figure 1:**
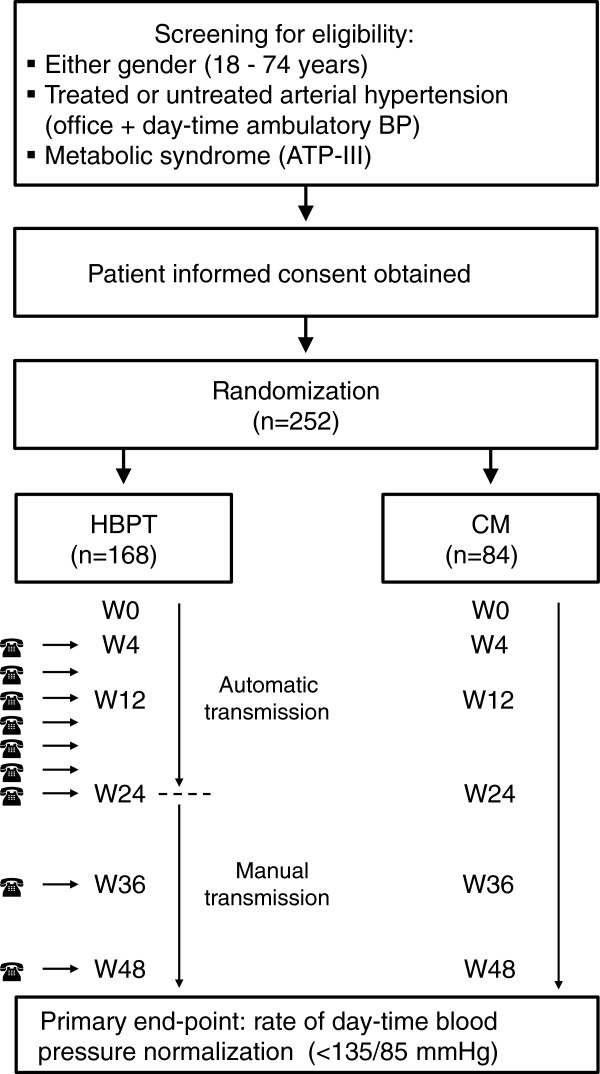
**Flow chart of the TELEBPMET study design.** ATP, Adult Treatment Panel; BP, Blood pressure; CM, Conventional management; HBPT, Home blood pressure telemonitoring; W, Week.

The study has an open label, parallel group, randomized, controlled design. After 1 week of run-in, patients will be randomized to two different arms: (1) intervention group (HBPT), with automated teletransmission of home blood pressure values to the Investigator, every month, over the first 24 weeks, followed by manual transmission of self-measured blood pressure data to the HBPT center immediately before each office visit, with regular doctor’s visit every 3 months, in the second 24 weeks (see below for details); (2) control group (CM) managed only through regular office visit every 3 months. A flow chart of the study design is reported in Figure 1.

### Study sites and population

This is an Italian, multicenter trial involving 12 Hypertension Centers, of which seven are located in northern Italy, two in central Italy, and three in southern Italy. The study will be conducted according to Good Clinical Practice guidelines and the Declaration of Helsinki. The protocol will be approved by the Ethics Committees of the centers involved.

The study will include hypertensive patients with metabolic syndrome, who are at an increased risk of cardiovascular events in the short term, and for whom adequate blood pressure control and treatment adherence are clinically relevant issues.

In order to be eligible for inclusion in the study the patients have to comply with the following criteria: (1) male or female gender and age between 18 and 74 years; (2) untreated or treated, but uncontrolled, essential arterial hypertension (office systolic blood pressure ≥140 mmHg and/or diastolic blood pressure ≥90 mm mmHg plus average daytime ambulatory systolic blood pressure ≥135 mmHg and/or average daytime diastolic blood pressure ≥85 mm Hg); (3) presence of metabolic syndrome according to ATP-III criteria [22], namely occurrence of at least two of the following four clinical conditions: abdominal obesity (waist circumference ≥102 cm in men and ≥88 cm in women), raised triglycerides (≥150 mg/dL or under specific treatment with fibrates or nicotinic acid), reduced HDL cholesterol (<40 mg/dL in men and <50 mg/dL in women or under specific treatment with fibrates or nicotinic acid), elevated fasting glucose (≥100 mg/dL or use of medication for hyperglycemia).

Main exclusion criteria are: (1) secondary hypertension; (2) renal or liver impairment; (3) severe autoimmune diseases; (4) neoplasia; (5) atrial fibrillation, frequent ectopic beats or severe conduction disturbances (second and third degree atrio-ventricular block), which may make automatic blood pressure measurement difficult or unreliable; (6) an arm circumference >32 cm or <22 cm; (7) any other condition that might compromise the patient’s participation in the study, including illiteracy and diseases preventing the completion of questionnaires (for example, myopia); (8) patients not able to follow study procedures.

Patients meeting inclusion criteria will be fully informed about the study design and purposes and asked to give a written informed consent, if willing to participate. After obtaining the written informed consent, eligible patients will be randomized to one of the two study arms.

### Randomization

Randomization will be performed using a blocked randomization procedure (computerized random numbers), in order to obtain equal numbers in each study arm. Patients will be randomized to CM or HBPT (1:2). In order to prevent selection bias, the allocation sequence will be concealed from the Investigator enrolling and assessing patients in sequentially numbered, opaque, and sealed envelopes.

### Description of the intervention: HBPT

At the time of the randomization, patients assigned to HBPT, will be equipped with an electronic blood pressure measuring device and a wireless transmitting interface. These devices will be kept at home until the end of the study.

The patient will be adequately trained by the Investigator on measurement procedures. Patients will be asked to self-measure their blood pressure at home twice a day, using a validated, automatic, electronic, oscillometric, upper arm monitor (A&D UA-767PC, A&D Company Ltd., Tokyo, Japan) [23]. The device can store up to 126 blood pressure measurements in its memory, is operated by non-rechargeable batteries, and is equipped with a serial port for interfacing with a personal computer or a transmission unit.

Two measurements, at a 2-min interval, will be taken in the morning before going to work or before starting daily activities (between 08:00 and 10:00), and two measurements will be taken in the evening (between 18:00 and 22:00), for a minimum of 3 days a week. The measurements have to be taken in a quiet environment, with the patient in a relaxed, comfortable, sitting position for at least 5 min before self-measurement.

After each measuring session and when not used for measuring blood pressure, the blood pressure monitor will be connected through a serial cable to a GPRS wireless interface (BluMed). During the first 24 weeks, the transmitter will send blood pressure data every day to the http://www.morepress.net website, (Biotechmed Ltd., Somma Lombardo, Varese, Italy) where they will be analyzed and a report will be generated and sent once a month via email to the Investigator. A feedback on the status of patient’s blood pressure control will also be visualized on the interface display.

The wireless interface also comes with a button which allows the patient to manually send the data to the HBPT center whenever it is deemed necessary (for example, in case of symptoms or very high blood pressure values), independently from the scheduled program. During the last 24 weeks of the follow-up no automatic data transmission will be done, but the patient will be requested to manually send to the HBPT center the self-measured blood pressure data, immediately before each scheduled office visit.

**Figure 2:**
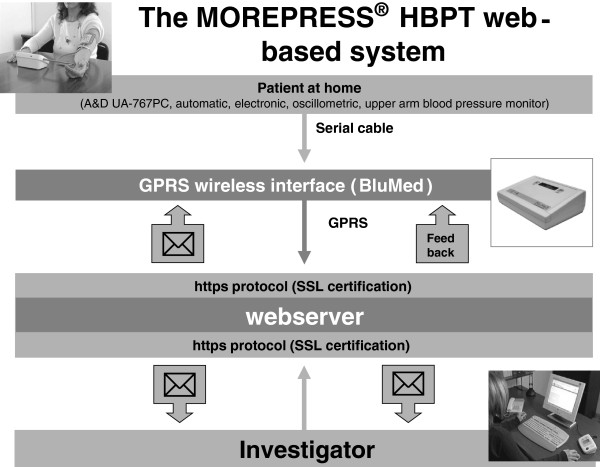
**The Morepress web**-**based telemedicine system used in the TELEBPMET Study.** GPRS, General Packet Radio Service; HBPT, Home blood pressure telemonitoring; SSL, Secure Sockets Layer.

A diagram of the telemonitoring web-based system is shown in Figure 2.

### Description of the control condition

In the CM or control group, blood pressure will be measured only during the office visit by the same automatic device used by patients of the HBPT randomization group. After 5 min sitting, automated blood pressure measurements will be performed twice at a 2-min interval and the average of the measurements will be calculated and used as clinical reference.

### Study procedures

**Figure 3:**
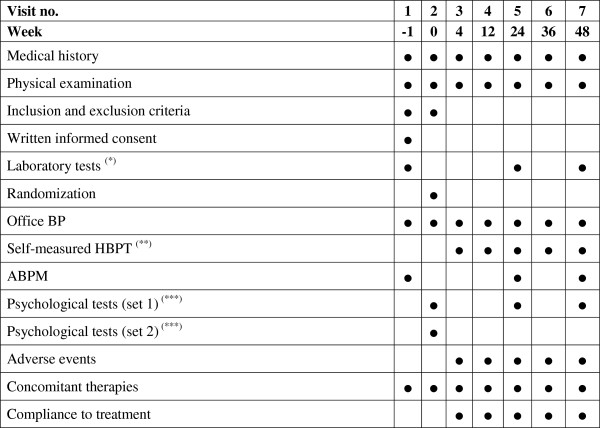
**Timing and content of study assessments.** ABPM, Ambulatory blood pressure monitoring; BP, Blood pressure; HBPT, Home blood pressure telemonitoring.

Besides baseline screening and randomization visits (Visit 1 and 2), all the patients will be re-assessed at 4 weeks (Visit 3), 12 weeks (Visit 4), 24 weeks (Visit 5), 36 weeks (Visit 6), and 48 weeks (Visit 7) of follow-up. If the Investigator deems it appropriate extra visits can be planned. Figure 3 presents an overview of variables measured at each time point of the study. All data collected during the study will be entered manually by the Investigator in a web-based electronic case report form (e-CRF) linked to the telemedicine system.

The study includes a first visit at the Hypertension Center (Visit 1) where automatic office blood pressure is measured, the patient’s history gathered, a physical examination performed. Laboratory tests useful for the determination of the organ damage and cardiovascular risk will be assessed as well. These tests include biochemistry and urinalysis (plasma lipids, fasting glucose, glycosylated hemoglobin, creatinine, C-reactive protein, albumin/creatinine ratio on morning urine sample or 24-h microalbuminuria), an echocardiogram with determination of left ventricular mass, a carotid ultrasonography for determination of intima-media thickness, a fundoscopy, and an electrocardiogram (ECG). Waist circumference will also be measured. A 24-h ambulatory blood pressure monitoring will be performed (see below for details).

Within 1 week from Visit 1 those patients complying with the inclusion criteria (that is, values of office systolic blood pressure ≥140 mmHg and/or office diastolic blood pressure ≥90 mmHg plus daytime systolic blood pressure ≥135 and/or daytime diastolic blood pressure ≥85 mmHg, plus presence of metabolic syndrome) will be randomized to the CM group or to the HBPT group (Visit 2).

During the same visit the patients will be administered the following psychological tests: Medical Outcome Survey Short-Form 36 (SF-36), Psychological General Well-Being Index (PGWBI), Beck Depression Inventory (BDI), Locus of Control (LoC), Self-Esteem Inventory (SEI), Illness Behaviour Questionnaire (IBQ) and Coping strategies (COPE), forming set 1, and Trait Anxiety Inventory (STAY), Type D Personality (TDP), Type A Personality (TAP), Temperament and Character Inventory (TCI), forming set 2 [24-35]. In untreated patients an antihypertensive treatment will be initiated. In treated patients, antihypertensive drug treatment will be adjusted in order to improve blood pressure control and possibly achieve office blood pressure normalization (<140/90 mmHg) on the next visit.

Four weeks later (Visit 3) the patients will be visited again by the Investigator and the medical therapy will be adjusted on the basis of office blood pressure (target, <140/90 mmHg) in the CM group or home blood pressure (target, <135/85 mmHg) in the HBPT group. Office blood pressure will still be measured by the Investigator with the same automatic electronic instrument used for self-measurement, in all patients, including those randomized to HBPT. Additional visits are scheduled 12, 24, 36, and 48 weeks after randomization (Visits 4, 5, 6, and 7). During each of these visits office blood pressure will be measured and antihypertensive medication will be changed according to office (CM group) or home blood pressure values (HBPT group). Drug treatment can also be changed in the patients of the HBPT between the scheduled visits, based on the home blood pressure report monthly received and reviewed by the Investigator. The same laboratory tests performed at screening visit and a 24-h ambulatory blood pressure recording will be repeated at Visits 5 and 7. During these two visits psychological tests will also be administered, with the exclusion of STAY, TDP, TAP, and TCI test, administered only at Visit 2.

Occurrence of adverse events, adherence to therapy and concomitant therapies will be checked at all visits during the trial. Evaluation of adherence to drug treatment will be based on patient’s report on amount of prescribed drug intake and on counting the quantity of study medication taken by the patient, comparing this datum with the quantity expected to be returned. The Investigator will instruct each patient to bring back used and unused drug blisters at each visit. The compliance will be computed as the ratio between the number of pills taken and the number of pills which should have been taken, expressed as a percentage, and will then be graded as follows: excellent (100% of prescribed pills taken), good (80% to 99%), fair (40% to 79%), or poor (<40%).

### Ambulatory blood pressure monitoring

At Visit 1, and subsequently at Visit 5 and at final visit (Visit 7) an ambulatory blood pressure monitoring will be performed non-invasively over 24 h by an oscillometric validated device [36,37]. Current Guidelines will be followed for proper recording performance [38,39]. The monitoring cuff will be wrapped around the non-dominant arm and the patient will be asked to keep her/his arm still during the automatic blood pressure measurements. The device will be programmed to measure blood pressure every 15 min during the whole 24 h. Each recording will start in the morning, before 07:00 and 10:00, immediately after office blood pressure assessment. Patients will then be sent home, asked to resume normal life, and to come back 24 h later for removal of the device.

### Home blood pressure monitoring

The modality of self-blood pressure measurement at home has been detailed in the previous sections. In the HBPT group home blood pressure reports will be automatically transmitted to the Investigator every month for the first 12 weeks (from Visits 2 to 5). Based on these reports, the Investigator will be allowed to adjust drug treatment before the next visit or to schedule an extra study visit. Starting from Visit 5 until the end of the study (Visit 7), the patients randomized to HBPT will continue to self-measure blood pressure at home, but automatic transmission will be disabled and each patient will be asked to manually send blood pressure data the day immediately preceding the office visit at the Center. These home blood pressure measurements will be reviewed by the Investigator exclusively on Visits 6 and 7, and treatment adjusted accordingly.

### Psychological tests

With the aim to investigate the psychological impact of HBPT clinical psychologists will assess psychological dimensions at Visits 2, 5, and 7 by using the following standardized questionnaires: 

(1) SF-36: a generic tool for assessing the quality of life, aiming at quantifying the impact of disease on general health and on the psychological profile, for the determination of the most characteristic aspects affecting quality of life in relation to a standard reference population [24]

(2) PGWBI: a tool for the assessment of psychological wellbeing [25]

(3) BDI: this test can be considered as a standard reference among the self-assessment scales. A key feature of the scale is the precise definition of the criteria for quantification, since every item corresponds to a given level of severity. The scale is the most specific self-assessment tool for depression, exploring a narrow range of symptoms by excluding those related to anxiety [26]

(4) LoC: this test measures the tendency to attribute the causes of behavior to internal or external conditions [27,28]

(5) SEI: this test measures the degree of self-esteem of the patient [29]

(6) IBQ: this test measures the disease perception [30]

(7) COPE: the test is designed to be a measuring instrument capable of assessing the more subtle individual differences in coping. It is able to balance the general trend of the subject with the current response to the stressful situation. Due to its characteristics, this test is suitable for studies aiming at clarifying more thoroughly the possible influence of personality aspects on adaptation [31]

In order to investigate the personality traits underlying the adherence behavior, clinical psychologists will also assess the following psychological dimensions only at Visit 2 by using the following standardized questionnaires:

(1) STAY: this test assesses the degree of trait anxiety severity [32]

(2) TDP: the test evaluates the presence of the personality traits defined as being distressed [33]

(3) TAP: the test evaluates the presence of type A personality traits [34]

(4) TCI: a questionnaire for the self-evaluation of different personality characteristics, normal and abnormal, which is based on an interpersonal theory of personality [35]

All psychological questionnaires used in the study are psychometrically validated and standardized on the Italian population.

### Statistical analysis

#### Primary outcome measures

The primary study endpoints are (1) the percentage of patients achieving daytime blood pressure normalization (systolic blood pressure <135 mmhg and diastolic blood pressure <85 mmhg) 24 weeks after randomization (visit 5); and (2) the percentage of patients achieving daytime blood pressure normalization after 48 weeks (visit 7).

The primary outcomes will be separately assessed for the HBPT and CM groups.

#### Secondary outcome measures

A series of psychological and clinical variables will be used as secondary endpoints.

Scoring of the different psychological questionnaires will be computed for the HBPT and CM groups and compared between groups. They will be used to quantify therapeutic adherence, and compared with evaluation of compliance based on the patient’s report.

The following clinical endpoints will also be evaluated: (1) the percentage of patients with office blood pressure normalization (<140/90 mmHg) after 24 and 48 weeks; (2) percentage of patients with home blood pressure normalization (average <135/85 mmHg) after 24 and 48 weeks in the HBPT group; (3) changes in average 24-h systolic and diastolic blood pressure after 24 and 48 weeks *vs*. baseline; (4) changes in office systolic and diastolic blood pressure after 24 and 48 weeks *vs*. baseline; (5) changes in average home systolic and diastolic blood pressure after 24 and 48 weeks *vs*. baseline in the HBPT group; (6) the number of antihypertensive drugs prescribed after 24 and 48 weeks *vs*. baseline; (7) the number of office or phone visits after 24 and 48 weeks *vs*. baseline; (8) direct costs of patient management, based on additional medical visits and laboratory tests performed between baseline and 24 and 48 weeks; (9) adherence to treatment, as reported by the patient (the assessment of adherence and persistence to therapy will also be done indirectly, by evaluating adherence to the home blood pressure monitoring program and by evaluation of the psychological tests); (10) difference in target organ damage score between baseline and week 24 and 48 of the study; (11) difference in cardiovascular risk level between baseline and week 24 and 48 of the study according to Heart Score (ESH-ESC) and Framingham algorithm [40,41]; (12) difference in the rate of cardiovascular events between baseline and 24 and 48 weeks of follow-up; and (13) the number of subjects showing regression of metabolic syndrome after 24 and 48 weeks from the randomization.

#### Sample size

The underlying study hypothesis is that the percentage of subjects achieving daytime blood pressure normalization will be 25% larger in the HBPT group. Using a two-tailed test with a 0.05 significance level and an 80% power, the estimated number of patients to be randomized is 252 (including a 25% drop-out rate), with 84 patients randomized to CM and 168 to HBPT.

#### Planned statistical analyses

Analysis will be performed on patients valid for intention-to-treat, defined as all subjects with at least one visit during the randomized phase of the study and with valid ambulatory blood pressure recordings at baseline, after 24 and 48 weeks of follow-up even in the presence of protocol violations. A confirmatory analysis on the per-protocol (patients who have successfully completed the study without major protocol violations) is also foreseen.

The demographic and clinical characteristics of the two study groups will be compared at baseline by analysis of variance (continuous variables) or by Mantel-Haenszel Chi-square test (categorical variables), in order to verify the between-group homogeneity at randomization. The same tests will be used to compare primary and secondary study endpoints between the CM and HBPT groups. If deemed necessary, in addition to the univariate approaches, multivariate analysis methods will be used.

The type and frequency of drug-related adverse events will be reported for each randomization group. The absolute rate of adverse events will be compared between groups using the Chi-square test.

The minimum level of statistical significance will be set at *P* <0.05.

## Discussion

This article describes the background, objectives, and design of a randomized controlled trial that will test the superiority of HBPT as compared to CM in improving blood pressure control in high-risk hypertensive patients with metabolic syndrome. Our study will help adding further evidence on the effectiveness of treatment decision of hypertension based on home blood pressure monitoring or HBPT. Findings from previous studies are controversial to this regard. In the THOP (Treatment of Hypertension based on home or Office blood Pressure) trial [42], antihypertensive drug treatment in hypertensive patients with an office diastolic blood pressure ≥95 mmHg, was adjusted in a stepwise fashion based on either the self-measured diastolic blood pressure at home or on readings taken at the doctor’s office. Adjustment of antihypertensive treatment based on home blood pressure led to less intensive drug treatment and marginally lower costs, but also to less blood pressure control, with no differences in quality of life and target organ damage. Similarly, in the HOMERUS (Home versus Office blood pressure MEasurements: Reduction of Unnecessary treatment Study) trial [43] self-measurement of blood pressure at home led to less medication use than office blood pressure measurement without leading to significant differences in office blood pressure values or target organ damage. However, in both the aforementioned studies, the target value for home blood pressure was the same as that for office blood pressure and higher than that recommended by current guidelines [9,39]. More recently, the HOMED-BP (Hypertension Objective Treatment Based on Measurement by Electrical Devices of Blood Pressure) [44] proved that adjusting antihypertensive drug treatment on the basis of blood pressure values collected through HBPT is feasible and effective for maintaining an optimal target blood pressure level and optimal antihypertensive medication. This is in line with a systematic meta-analysis of randomized controlled studies, which demonstrated the superiority of HBPT *vs*. usual care in improving blood pressure control in either low- or high-risk hypertensive patients [21].

Secondarily, the trial will investigate and compare between randomization groups personality traits predictive of adherence. This will be useful to identify possible psychological determinants of adherence to antihypertensive treatment, also relating them to blood pressure response to antihypertensive treatment in daily life conditions. Poor adherence to treatment remains one of the major limitations in the management of hypertension: it may cause poor blood pressure control and may contribute to increased morbidity, mortality, and costs [45,46].

Previous studies have shown that persistence with antihypertensive therapy decreases with time: discontinuation rates vary from 22% to 50% of patients during the first year after treatment initiation [47-50]. Therefore, improving adherence to treatment remains a major challenge for the treating physician. Use of blood pressure telemonitoring technique, which might help in improving blood pressure control and adherence, together with quantification and definition of the psychological trait of the hypertensive patient, might help identifying features of high-risk hypertensive patients and increase the chance of successful treatment.

The study will also evaluate the impact of HBPT on additional medical and economic outcomes, which might help in unmasking possible additional advantages of this technique in high-risk hypertensive patients, on a relatively long-term follow-up.

Before drawing the conclusions we must acknowledge that the study protocol suffers from some potential methodological limitations. First, allocation of patients to one of the two study groups will be concealed from the Investigator by means of sequentially numbered, opaque, and sealed envelopes. We acknowledge that this approach may introduce a bias and that central computerized randomization would be preferred. However, web-based randomization was not possible due to limited availability of continuous Internet connection in some centers and telephone interactive voice response services could not be afforded because too expensive. Second, assessment of medication adherence will be based on both self-report of patients and on manual pill count by the Investigator. This methodology might be imprecise. However, adherence is not the primary study endpoint, and though the use of electronic pill-box monitoring was taken into account at the time of study planning and designing, we opted out of this choice because the device was too expensive and its management too complex for the study purposes.

## Conclusions

At the moment no studies have assessed the impact of HBPT and of the quantification of patient’s psychological characteristics on improvement of blood pressure control and adherence to antihypertensive treatment. The TELEBPMET Study will show whether this approach is effective in management of hypertensive patients with metabolic syndrome. It will also provide a comprehensive understanding of the psychological determinants of medication adherence and blood pressure control in high-risk patients with hypertension.

## Trial status

The TELEBPMET study trial was conceived and designed in 2008. At the time this manuscript was submitted full approval by the Medical Ethics Committee has been obtained for all centers and recruitment was started. The patients’ follow-up is currently ongoing.

## Abbreviations

ATP: Adult Treatment Panel; BDI: Beck Depression Inventory; CM: Conventional management; COPE: Coping Orientation to Problems Experienced; e-CRF: Electronic Case Report Form; ECG: Electrocardiogram; ESC: European Society of Cardiology; ESH: European Society of Hypertension; GPRS: General Packet Radio Service; HBPT: Home blood pressure telemonitoring; HDL: High density lipoproteins; IBQ: Illness Behavior Questionnaire; LoC: Locus of Control; PGWBI: Psychological General Well Being Index; SEI: Self-Esteem Inventory; SF: Short-Form; STAY: State-Trait Anxiety Inventory; TAP: Type A Personality; TCI: Temperament and Character Inventory; TDP: Type D personality.

## Competing interests

GP has received honoraria for lectures by Omron and Microlife companies, manufacturers of home blood pressure devices. SO is Scientific Consultant of Biotechmed Ltd., manufacturer of the Morepress telemedicine system used in this study. EG is Medical Director of Bracco Pharmaceuticals, the company sponsoring the project. All the other authors declare that they have no competing interests.

## Authors’ contributions

GP, SO, and AC conceived the study. All authors participated in the trial design. SO and AC wrote the first draft of this manuscript. All authors commented on drafts of this paper, and read and approved the final manuscript.

## Authors’ information

The Telebpmet Study Group

## Coordinator

Gianfranco Parati (Milano)

## Data management

Stefano Omboni (Varese), Tiziana Gazzola (Varese), Edward Callus (Milano)

## Investigators

Milano: Gianfranco Parati, Laura Lonati, Francesco Della Rosa, Ellen Tosazzi

Varese: Achille Venco, Anna Maria Grandi, Andrea Maria Maresca, Christian Mongiardi, Micaela Mare

Broni: Maurizio Destro, Alessandra Rossi Ricci, Francesca Cagnoni, Joannhe Georgatos, Valeria Besostri

Pavia: Giuseppe Villa, Viviana Ferrari, Ombretta Omodeo

Padova: Paolo Palatini, Francesca Dorigatti, Elisa Bonso, Chiara Guarnieri

Brescia: Enrico Agabiti Rosei, Maria Lorenza Muiesan, Anna Paini, Deborah Stassaldi

Lumezzane: Simonetta Scalvini, Angelo Cinelli, Palmira Bernocchi, Silvana Rocchi

Pisa: Stefano Taddei, Armando Magagna, Lorenzo Ghiadoni, Irene Del Frate, Francesca Boresi, Antonella Guidi

Roma: Dario Manfellotto, Maria Antonietta Re, Luisa Pellicciotti, Antonia Florio, Giuliana Morani

San Felice a Cancello: Carmine De Matteis, Silvana Di Lillo, Antonietta Ambrosio

Salerno: Michele Guglielmi, Antonio Casciello, Maria Quaglia

Bari: Stefano Favale, Cinzia Forleo, Maria Annunziata Ardito, Stefania Gerunda, Mariligia Panunzio

## Scientific Committee

Gianfranco Parati (Milano), Stefano Omboni (Varese), Angelo Compare (Bergamo), Enzo Grossi (Milano)
